# 肺癌相关治疗与间质性肺病

**DOI:** 10.3779/j.issn.1009-3419.2013.05.09

**Published:** 2013-05-20

**Authors:** 时 金

**Affiliations:** 150081 哈尔滨，哈尔滨医科大学附属肿瘤医院肿瘤内科 Department of Medical Oncology, Tumor Hospital Affiliated to Harbin Medical University, Harbin 150081, China

**Keywords:** 肺肿瘤, 间质性肺病, 放疗, 化疗, 靶向治疗, Lung neoplasms, Interstitial lung disease, Radiotherapy, Chemotherapy, Targeted therapy

## Abstract

间质性肺病是肺癌相关治疗引起的严重副反应之一，因诊断的复杂性及病情的多变性往往不能及时诊治甚至危及患者的生命，严重影响患者的预后。放、化疗及靶向治疗导致间质性肺炎的机制及相关因素尚不完全清楚。因此，如何减少、早期发现及诊治肺癌相关治疗引起的间质性肺病成为今后肺癌治疗过程中不可忽略的一个问题。

## Abstract (en)

Interstitial lung disease is one of the most serious side effects caused by lung cancer treatment. Due to the complexity diagnosis and variability of the disease, it is often not diagnosied and treated in time, even endanger the patient's life and affect the patient's prognosis. The mechanism of interstitial pneumonia caused by radiotherapy, chemotherapy and targeted therapy is still not fully known. Therefore, it become a problem that how to early detection, diagnosis and treat the interstitial lung disease caused by lung cancer treatment and it should not be ignored in the future.

肺癌是当前全球范围内最常见的恶性肿瘤之一^[1]^，肺癌的治疗手段主要包括手术、化疗、放疗及靶向治疗等^[2]^，然而在治疗的同时，不可避免地会产生各种相关的副反应，间质性肺疾病（interstitial lung disease, ILD）是放、化疗及靶向治疗的主要副反应之一。ILD是以弥漫性肺实质、肺泡炎和间质纤维化为基本病理改变，以活动性呼吸困难、X线胸片示弥漫阴影、限制性通气障碍、弥散功能降低和低氧血症为临床表现的不同类疾病群构成的临床病理实体的总称，范围从良性浸润到致命性急性呼吸窘迫综合征。由于化疗、放疗及靶向治疗相关的ILD是一个不可预知、发展迅速、诊断困难的疾病，因此严重影响患者预后甚至危及患者生命。如何系统地评估与ILD相关的因素，预防、诊断及治疗ILD是必要的。本文系统综述了肺癌相关治疗与ILD的关系。

## 1 概述

ILD这一病名的提出早在1975年第18届Aspen肺科讨论会时使用了这一名词。时隔10年（1985年）第28届Aspen肺科讨论会再一次对ILD做了研讨。经过数十年的发展，对ILD的研究已有很大的进展。间质性肺疾病是一组主要累及肺间质、肺泡和细支气管的肺部弥漫性疾病，通常亦称作弥漫性实质性肺疾病（diffuse parenchymal lung disease, PLD）。ILD并不是一种独立的疾病，它包括200多个病种。尽管每一种疾病的临床表现、实验室和病理学改变有各自的特点，然而，他们具有一些共同的临床、呼吸病理生理学和胸部X线特征，表现为渐进性劳力性气促、限制性通气功能障碍伴弥漫性功能降低、低氧血症和影像学上的双肺弥漫性病变。病程多缓慢进展，逐渐丧失肺泡-毛细血管功能单位，最终发展为弥漫性肺纤维化和蜂窝肺，导致呼吸功能衰竭而死亡^[3]^。大约20%-30%的非小细胞肺癌患者^[4]^及4.5%的小细胞肺癌患者被诊断为肺癌时发现同时患有间质性肺病^[5]^。近年来，随着医疗技术水平的不断进步，化疗药物的研发、放疗技术的提升、靶向药物的诞生使得肺癌的治疗手段也逐渐多样化，肺癌患者的生存期较前明显延长。然而，在看到希望的同时，放、化疗及靶向治疗所带来的副作用，尤其是间质性肺病的发生严重影响患者的预后，成为今后治疗过程中不可忽略的一个问题。

## 2 肺癌相关治疗与ILD

### 2.1 化疗与ILD

化疗是利用化学药物杀死肿瘤细胞，抑制肿瘤细胞的生长繁殖和肿瘤细胞的分化，是一种全身性的治疗手段。目前化疗仍是肺癌尤其是晚期肺癌最主要治疗手段，即便是早期接受一线手术治疗的患者，术后辅助化疗仍能增加5%的5年生存率^[2]^。但是化疗在杀伤肿瘤细胞的同时，也会对正常细胞及组织产生损害，其中化疗药物导致的肺毒性是化疗药物严重的副反应之一。“化疗肺”是指在化疗周期中或化疗后出现的由抗肿瘤药物引起的气管、支气管、肺泡等肺部损伤的一组疾病，主要累及肺间质，可出现间质性炎症、肺泡出血及肺泡弥漫性损伤等，病情变化迅速，可短期进展至呼吸衰竭或急性呼吸窘迫综合症。大多数化疗药物均可能引起“化疗肺”，如：抗生素类（博来霉素、丝裂霉素）、烷化剂类（白消安、环磷酰胺）、抗代谢类（甲氨蝶呤、吉西他滨）、亚硝胺类（亚硝基脲）和鬼臼毒素（紫杉醇、多西紫杉醇）等^[6]^。大多数肺间质损伤通常出现在化疗开始几周之内，称之为急性ILD，其病理特点主要表现为弥漫性肺泡损伤（diffuse alveolar damage, DAD），由于诊断的复杂性，常常不能获得及时准确的估计和治疗，有些则相对较晚（> 化疗后2个月），导致肺纤维化后遗症^[7]^。

与化疗引起间质性肺炎相关的因素主要包括以下几个方面：①化疗药物的种类，尤其第三代化疗药物如吉西他滨、多西他赛相比其它药物发生ILD的风险明显增高，在一项最新的392例患者参与的多西他赛单药化疗的临床实验中，18例患者化疗后发生ILD（4.6%）^[8]^。而Okuda^[9]^的报道则指出长春瑞滨联合铂类治疗合并间质性肺炎的非小细胞患者仍然是安全有效的；②化疗药物的剂量及方案的选择，Shukuya^[10]^的相关研究中发现，虽然单周紫杉醇方案相比三周一次的紫杉醇方案毒性有所减低，但是会加快药物对肺毒性的频率，因此，发生ILD的几率反而要比三周一次的用药方案更大；③之前有过肺部放疗史的患者与ILD发生率密切相关，Umemura^[11]^在一项118例患者接受吉西他滨化疗发生ILD的研究中发现，之前有过肺部放疗史的8例患者中有3例发生ILD（37.5%），而之前没有放疗史的110例患者中有6例发生（5.5%）（*P* < 0.05）；④之前有肺纤维化的患者化疗后发生间质性肺炎的几率明显增加，在Kenmotsu等^[12]^的研究中发现之前有肺纤维化的患者化疗后发生ILD的风险与之前没有IPF的患者相比为30%:8%。而且，Togashi^[13]^的研究中指出之前存在ILD的患者与无ILD的患者化疗后总生存期相比为17.8个月:10.7个月（*P*=0.001）；⑤其它，如使用粒细胞集落刺激因子或肺水肿等因素。

### 2.2 放疗与ILD

放射治疗无论在肺癌的治愈性治疗或姑息性治疗过程中均起到重要作用，尤其随着放射技术及设备的不断进步，放射治疗的作用越来越重要。同样，放射性治疗也是一把双刃剑，放射治疗在控制肿瘤病灶的同时，不可避免的会损伤肿瘤周围的区域。放射性肺损伤是指放射治疗在控制肿瘤病灶的同时可使肿瘤临近的肺组织因受到的放射剂量超过其发生生物效应的域值而产生不同程度的肺损伤^[14]^。其病理改变是一个动态发展过程，随着照射时间的延长逐渐加重，肺泡是主要损伤部位，基本病变为肺充血、水肿、肺间质增厚及肺泡腔萎陷变小。一般认为放射性肺损伤有两种表现形式，即早期的急性放射性肺炎和后期的放射性纤维化。我们通常所说的放射性间质性肺炎是指后期的放射性纤维化^[15]^。放射性ILD的分子机制尚不完全清楚，一种说法是放射线破坏细胞的结构和大分子如脂类、肽类或者脱氧核糖核苷酸，细胞处于快速的代谢状态而使发生纤维化风险增高。另一种说法是放射线引起的巨噬细胞释放促炎性细胞因子如IL-6、TGF-β等进一步促进间质性肺炎的形成。

与放射性肺炎相关的因素主要包括以下几个方面：①放射剂量的多少，提高剂量虽然可以提高病灶控制率，但是随着放射剂量的不断增加发生放射性肺炎的几率也明显增高。三维适形放疗（three dimensional conformal RT, 3D-CRT）和调强放射治疗（intensity-modulated radiation therapy, IMRT）的出现解决了这一问题，使放疗从二维时代进入了三维时代。三维适形放疗通过调整放射野形态使之与肿瘤区尽可能在三维空间达到一致，从而减少正常组织的损伤。为放射剂量提高提供了基础。Belliere^[16]^的研究中，50例非小细胞肺癌患者行3D-CRT放疗，结果35例（70%）的患者接受了74 Gy的剂量，而心、肺毒性均在耐受范围内；②放射野的大小，如将30 Gy分次照射双肺面积25%的区域则不会引起任何症状，然而，如果同样的剂量照射双肺全部的面积后果很可能是致命的。近年，随着四维放疗技术的应用逐渐增多，使得放射靶区更加精确，减少不必要照射区域的损伤。图像引导放射治疗（image guided radiation therapy, IGRT）是在三维适形放疗的基础上引进了时间的概念，通过监测靶区在放疗中的空间位置、体积大小和放疗剂量分布的变化来实现照射过程中对靶区位置及放疗剂量的实时调整。尤其是配合4D-CT^[17]^和呼吸门控技术的自适应IGRT，使其在线校准、在线调整放疗计划等技术进一步完善。Harsolia等^[18]^应用IGRT技术配合4D-CT治疗8例肺癌患者，结果显示计划靶区（planning target volune, PTV）体积较3D-CRT平均缩小约44%，充分体现了IGRT的优势。同样，在看到IGRT优势的同时，也要注意到IGRT不利的一面，IGRT需要利用电子射野影像系统进行图像采集，相应会导致1%-2%的额外辐射，其次IGRT延长了治疗时间^[19]^。近年，随着容积弧形调强放射治疗技术（volumetric modulated arc therapy, VMAT）的诞生使得IGRT进一步完善和优化，VMAT的优点在于可360°设定任何角度范围内旋转照射，同时可设定2个弧，可行非共面照射，从而保证治疗区剂量分布一致，VMAT通过持续地改变机架旋转速度、多叶准直器的位置、剂量率，来调整各个角度的剂量强度，在机架旋转的同时，不间断照射，从而缩短治疗时间，减少了治疗区运动，更提高了治疗精度。Holt^[20]^通过27例患者对比IMRT与VMAT的平均放疗时间为23.7 min:6.6 min。Otto^[21]^应用VMAT技术，单次剂量200 cGy的执行时间仅为1.5 min-3 min，相比IMRT缩短达75%-80%；③放疗计划及靶区的制定，靶区的制定直接关系到放射野的大小，近年，随着PET-CT的应用，使得靶区的勾画更加精确，PET-CT具有准确区分原发肿瘤病灶、鉴别转移淋巴结和发现远处转移灶等方面的优势，已经越来越多地应用于肿瘤放疗计划中^[22]^；④同时合并化疗或序贯化疗可能增加放射性肺损伤的风险。另外，突然停用化疗药物可能导致放射性ILD的发生称之为“回忆效应”^[23]^；⑤其它相关因素如PS评分、女性、老年等。

### 2.3 靶向治疗与ILD

肿瘤分子靶向治疗是指“针对参与肿瘤发生发展过程的细胞信号传导和其它生物学途径的治疗手段”。肺癌靶向治疗的相关研究已经达到一定水平，然而，在治疗的特异性以及有效性方面仍需要进一步深入研究，治疗过程中可能导致的毒副反应也不容忽视。靶向治疗导致ILD的机制尚不明确。已知EGFR参与肺部损伤的修复，推测EGFR-TKI通过抑制EGFR表达、减少组织中EGFR含量、降低EGFR的活性、抑制气道上皮细胞的生长和修复、妨碍肺部损伤的修复从而导致ILD。另有研究^[24]^发现靶向治疗相关间质性肺病患者支气管肺泡灌洗液中干扰素诱导蛋白10的水平增加，提示炎症反应可能参与了TKI诱导ILD的发生。Namba^[25]^的研究发现吉非替尼致ILD可能和吉非替尼抑制的热休克蛋白70（heat shock protein 70）的表达有关，也有推测ILD的发生可能与药物过敏、肿瘤坏死释放出大量的肿瘤坏死因子以及药物加重放射性肺炎等有关。尽管ILD发生机制不明，但多因素分析显示男性、吸烟、既往有肺纤维化、化疗史和一般状况差均是发生ILD的高危因素^[26]^。

肺癌靶向治疗中表皮生长因子受体酪氨酸激酶抑制剂领域的研究相对成熟，特别是吉非替尼，自2002年批准上市以来，经过相关的Ⅱ期实验已大量用于临床。但是在看到其对难治性非小细胞肺癌较好疗效的同时，其不良反应，尤其是致命性的间质性肺病的发生应同样引起重视，美国FDA的一项回顾性研究指出在50, 000例接受吉非替尼治疗的患者中发生ILD的风险为1%，其中吉非替尼导致ILD的发生率在日本大约2%，美国和其它地区大约为0.3%。在Nakagawa^[27]^的一项关于日本地区接受吉非替尼治疗的回顾性分析中发现，526例接受吉非替尼治疗的患者中，有17例（3.23%）发生ILD。而一项最新的台湾地区的回顾性分析显示1, 080例接受吉非替尼治疗的非小细胞肺癌患者中有25例（2.3%）患者出现ILD^[28]^，是否亚洲地区患者出现吉非替尼相关ILD的风险高于欧美地区？不同地区发病率差异的原因是什么？需要更大的样本分析及进一步科学调查。在另一项关于1, 900例日本非小细胞肺癌患者接受吉非替尼治疗的回顾性分析中指出经过4个月吉非替尼治疗后有70例（3.5%）患者发生ILD，其原因可能和*EGFR*突变患者的百分比或者患者的临床有效率相关。Kudoh等^[29]^的研究中发现，吉非替尼导致间质性肺炎的风险可能和年龄、性别、吸烟史、纤维化病史及PS评分有关。如果靶向治疗同时联合化疗，发生ILD的风险相对增加，尤其是联合吉西他滨时，因为两种药物均有较强的肺毒性。间质性肺疾病在接受另一种表皮生长因子受体酪氨酸激酶抑制剂厄洛替尼治疗的患者中同样被观察到^[30, 31]^，厄洛替尼相关的ILD和吉非替尼大致相似^[32]^，在一项关于3, 488例患者接受厄罗替尼治疗的回顾性分析中，ILD的发生率为4.5%（158例）^[33]^，相关的危险因素主要包括吸烟史、肺部感染、PS为2分-4分。ter Heine^[34]^的一项回顾性分析中指出：在既往报道的19例厄洛替尼相关的间质性肺病患者中，有11例患者厄洛替尼代谢物水平明显高于正常，考虑较高的血药浓度可能和厄洛替尼导致ILD相关。Soo-Jung^[35]^报道的1例50岁男患者，既往有吸烟史及化疗史，在应用厄洛替尼2天后便出现间质性肺炎症状，1周之后便死于因急性间质性肺炎导致的呼吸衰竭，所以对有高危险因素的患者要慎用厄洛替尼。

其它靶向治疗相关的ILD相对罕见，可能和新一代靶向药物作用靶点不同有关，不影响EGFR与肺部损伤的修复，另一方面也可能与样本量少有关，尚需要进一步探索。索拉菲尼为多靶点靶向药物，主要用于年龄较大的广泛期非小细胞肺癌，但目前的相关临床试验^[36]^尚未发现索拉菲尼相关ILD的副作用。另一项34例 > 70岁的非小细胞肺癌患者参与的伊马替尼联合紫杉醇治疗的Ⅱ期临床实验^[37]^中，也并未观察到ILD的发生。西妥昔单抗相关的肺毒性主要发生在结直肠癌肺转移的患者，Chua^[38]^报道了1例西妥昔单抗引起ILD的患者，患者在应用西妥昔单抗2个月后开始出现咳嗽、气短等症状，CT表现为毛玻璃样改变，虽经激素治疗后症状有所缓解，但最终死于进行性加重的呼吸衰竭。Achermann^[39]^的研究指出患者在应用西妥昔单抗5周-6周后，如果患者出现发热、气短，CT显示肺部毛玻璃样改变应警惕ILD的风险。

## 3 ILD的诊断及治疗

### 3.1 ILD的诊断

间质性肺病的诊断主要包括三个方面：临床症状，影像学检查，病理学检查。①临床症状：ILD的症状主要表现为胸闷气短、干咳、发热。化疗相关的ILD通常发生在治疗开始或治疗结束后几周之内，通常表现为短期内进行性加重的胸闷、气促，有些患者会伴随干咳或者发热^[40]^；放疗相关的ILD一般发生在放疗后6个月-24个月，典型的特征为逐渐加重的、不可逆的肺纤维化，很少有患者在放疗后6个月内发生肺纤维化，即便患者肺纤维化开始时症状较重，但是2年之内通常保持在稳定状态，因纤维化程度不同患者所表现的症状也轻重不一，有些患者表现为持久的乏力及肺部易感染，个别患者会出现中度的肺动脉高压，多伴随紫绀、气促、肝大及杵状指；靶向相关的ILD通常发生在开始口服靶向药物后3周-7周，主要表现为干咳、发热等症状，大约90%的吉非替尼相关ILD患者之前有化疗或放疗病史。临床上如果遇到肺癌患者口服吉非替尼期间突发的发热、干咳应警惕间质性肺炎的发生^[41]^，如果呼吸困难突然加重应警惕间质性肺炎致突发气胸的可能，及时监测胸部及各项生命体征，以免延误病情^[42]^；②影像学检查：绝大多数ILD患者X线胸片或CT显示双肺弥漫性阴影，阴影的性质可以是网格条索状、弥漫磨玻璃状、结节状，亦可呈现多发片状或大片状等，也可以混合存在。多数ILD可以致肺容积减少，后期可见区域性囊性病变（蜂窝肺），常伴肺容积的进一步减少。高分辨CT可以更细致的显示肺组织和间质形态的结构变化和大体分布特点，现已成为诊断ILD的重要手段^[43]^。De Ruysscher^[44]^的研究中发现在放疗开始后第7、14天评价肺部肿瘤的FDG-PET-CT摄取值可以预测放射性肺损伤的发生率，摄取值越高，放疗后发生放射性肺损伤的几率越高；③病理学检查：肺活检是诊断ILD的金标准，当病史、影像学检查及支气管肺泡灌洗等检查得不出推断性的诊断，必要时可以进行肺活检。肺活检分为两种，一种是应用纤维支气管镜或CT引导下穿刺肺活检，其优点为操作简便，安全性高，可作为常规检查，且便于复查，但因所取的肺组织过小，误诊率及漏诊率较高。另一种外科肺活检（包括胸腔镜或开胸肺活检），虽可全面观察肺泡炎的类型和程度，但此方法是有创性检查手段，对于肺癌患者来说是不必要的，所以临床很少应用。通常单一的检查办法很难确诊，需要结合病史、影像学及病理学综合考虑。

间质性肺病的上述症状并没有特异性，当肺癌病情进展时同样可出现上述症状，感染、心血管疾病或肺栓塞、慢性阻塞性肺疾病（chronic obstructive pulmonary disease, COPD）等疾病出现上述症状也很常见。如何与上述疾病鉴别也是临床工作中需要注意的问题。主要通过以下方法进行鉴别：①肿瘤进展：首先评价肿瘤标记物的水平，观察肿瘤标记物有无增高；其次进行影像学检查，通常影像学检查均能排除肿瘤是否进展，尤其注意有无胸腔积液或心包积液的出现；②肺感染：首先进行血常规检查，观察白细胞及中性粒细胞百分比等炎症指标是否高于正常值；其次进行痰培养及血培养，明确有无感染；最后C反应蛋白水平或聚合酶链反应等也有一定参考意义；③心脏疾病：首先进行心电图检查，观察是否有心肌缺血或心肌梗塞等；其次进行超声心动图检查观察心脏射血功能是否异常；最后观察有无心衰、心肌缺血等相应症状及体征；④肺栓塞：首先进行影像学检查，肺CT检查会显示肺动脉内低密度的充盈缺损，或核磁肺动脉造影；其次进行凝血功能检查及评估，尤其血浆D-2聚体敏感性较高，若其含量低于500 μg/L时有重要的排除诊断价值；再次可进行动脉血气分析，肺栓塞常表现为低氧血症、低碳酸血症，肺泡-动脉血氧分压差增大；最后，如以上检查不能确诊，可行肺动脉造影，属有创性检查；⑤慢性阻塞性肺气肿，首先COPD患者多有长期慢性病史，其次可进行肺功能检查，ILD肺功能检查以限制性通气障碍为主，肺活量及肺总量降低，残气量随病情进展而减少，换气功能往往在ILD早期可显示弥散功能明显下降。而在COPD患者通常表现为第一秒用力呼气容积减小，换气量减少，肺总量、残气量增高。

### 3.2 ILD的治疗

针对放化疗及靶向治疗引起的间质性肺病尚无固定的治疗方案，首先可选择撤药或停止放疗，大多数患者在停止导致ILD的相关治疗后间质性肺病的症状便会有所减轻。其次，应用糖皮质激素控制病情，如果患者ILD症状较轻，可给予患者口服地塞米松片或泼尼松片，或者静脉点滴糖皮质激素。如果患者症状较重可选择大剂量甲泼尼龙冲击疗法（250 mg，4次/d），3 d-5 d后改为常规剂量或口服，如减量过程中病情复发加重，应再重新加大剂量控制病情，仍然有效。应用糖皮质激素时应注意机会致病菌感染，结核患者需注意肺结核的复发，必要时联合应用抗结核药物，长期应用糖皮质激素应注意真菌的感染^[45]^。最后根据个体差异找出最佳维持量，避免复发。因特殊原因不能接受激素及不能耐受激素者可改用免疫抑制剂，或减少皮质激素量加用免疫抑制剂^[46]^。

## 4 结语

综上所述，间质性肺病是肺癌放化疗及靶向治疗所引起的严重副反应之一，因导致间质性肺病的机制多样化及临床症状与其它呼吸系统疾病相似，往往干扰病情的诊断，所以很多时候不能及时诊断，加之病情变化迅速，常常危及患者生命。因此，在临床工作中针对肺癌的相关治疗要提高警惕，早预防、早诊断、早治疗，减少间质性肺病及相关副反应的发生，从而进一步提高患者的生活质量及预后。
